# Nonlinear Strain Engineering via Meta‐Material Substrates for Flexible Sensors With Simultaneously Enhanced Sensitivity and Wide Sensing Range

**DOI:** 10.1002/gch2.70154

**Published:** 2026-09-08

**Authors:** Gyeongyeol Lee, Sung‐Wook Kim, Yong Whan Choi

**Affiliations:** ^1^ Korea Hydro & Nuclear Power Co., LTD. Gyeongju‐si Gyeongsangbuk‐do South Korea; ^2^ Department of Electrical and Electronic Engineering Silla University Busan South Korea; ^3^ Department of Aeronautical & Mechanical Engineering Silla University Busan South Korea

**Keywords:** crack‐based sensor, flexible strain sensor, kirigami structure, metamaterial, sensitivity–range trade‐off, strain engineering, wearable electronics

## Abstract

Flexible strain sensors face an unresolved trade‐off between sensitivity and sensing range. We present a meta‐material‐based sensor platform (meta‐sensor) that overcomes this by engineering local strain input to the sensing element. A strain‐stiffening curved spiral structure and a uniform‐stiffness kirigami‐patterned structure connected in series produce nonlinear strain partitioning: before mechanical transition, global strain concentrates at the sensor interface to amplify sensitivity; after transition, strain redistributes to extend sensing range. Integrating a kirigami crack‐based sensor (maximum applied strain range of 50%) onto this composite substrate extends the operational range to 125% applied strain‐a 2.5× range extension (from 50% to 125%) — while raising the mid‐range response to ≈3.5× that of a wide‐range reference sensor. The platform is validated in respiratory monitoring across rest and vigorous exercise, and knee rehabilitation tracking from early to late recovery. This meta‐material substrate approach offers a generalizable, material‐independent strategy for simultaneously achieving high sensitivity and wide sensing range.

## Graphical abstract

Strain sensors trade sensing range for sensitivity. A metamaterial substrate coupling strain‐stiffening and uniform‐stiffness architectures redistributes applied strain, so any bonded sensor experiences only a fraction of it. Demonstrated with a kirigami crack sensor, the platform extends the working range 2.5‐fold to 125% strain while retaining 3.5‐fold higher sensitivity, capturing respiration and knee flexion.

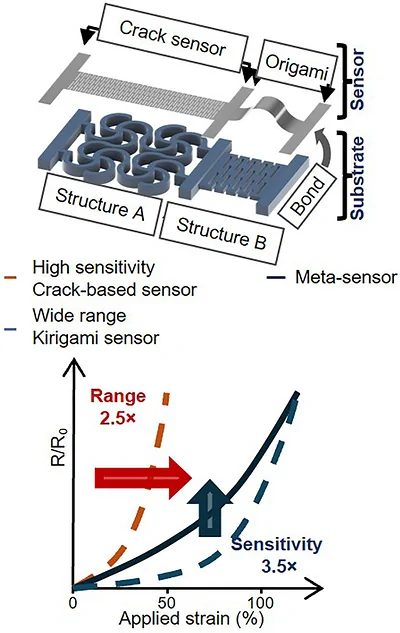

## 1 Introduction

Flexible resistive strain sensors have attracted considerable attention for applications in wearable health monitoring, soft robotics, and human–machine interfaces, owing to their mechanical compliance, lightweight form factor, and compatibility with biological surfaces [1, 2, 3, 4]. Among the available sensing mechanisms, crack‐based sensors have emerged as particularly promising candidates, offering exceptionally high gauge factors achieved through the controlled propagation and closure of micro‐cracks in metallic thin films deposited on polymer substrates [5, 6]. However, the crack geometry that confers high sensitivity also limits the operational strain range: at small strains, the cracks become fully open, and resistance exceeds measurable limits [5, 7]. Conversely, sensors designed to accommodate large strains — such as those incorporating kirigami‐patterned substrates [8, 9] — distribute the applied deformation across the structure, reducing the local strain at the sensing element and consequently lowering sensitivity [10]. This inverse relationship between sensitivity and sensing range is intrinsic to resistive sensing mechanisms broadly, and cannot be resolved by material substitution or geometric optimization of the sensing element alone [11, 12].

Prior work addressing this trade‐off has largely concentrated on the sensing material or sensor geometry itself — through the introduction of crack‐morphology engineering [13], hierarchical microstructures [14, 15], or heterogeneous composites [16]. These approaches share a common limitation: in all resistive sensing schemes, a higher rate of resistance change per unit strain corresponds to a smaller strain window before saturation, regardless of the material system. Incremental structural refinements of the sensing element therefore do not escape this constraint; they merely shift the operating point along the sensitivity–range frontier. Critically, none of these strategies addresses what is arguably the more tractable problem: the strain delivered to the sensing element is taken as a given, determined solely by the applied boundary conditions, rather than treated as a designable quantity.

Here, rather than accepting the strain input as fixed and attempting to extend the sensor's intrinsic response, we treat the local strain at the sensor interface as an engineering variable, controlled by the mechanical design of the substrate to which the sensor is bonded. To this end, we introduce a meta‐material substrate composed of two mechanically distinct sub‐structures in series: one exhibiting a well‐defined strain‐stiffening transition [17] and one maintaining constant modulus throughout deformation [8]. Their combination produces a nonlinear strain partitioning in which global strain is preferentially concentrated at the sensor interface while Structure A remains compliant, and progressively diverted away from the sensor once Structure A stiffens. Because the mechanism operates at the substrate level, it is independent of the choice of sensing material, and the target sensitivity and range profiles can be separately tuned through the structural parameters of each sub‐structure. A kirigami crack‐based sensor is used here as the representative sensing element to experimentally validate the concept, owing to its well‐characterized sensitivity–range trade‐off [5, 10] and relevance to wearable monitoring.

## 2 Results

### 2.1 Meta‐Sensor Architecture and Operating Principle

**FIGURE 1:**
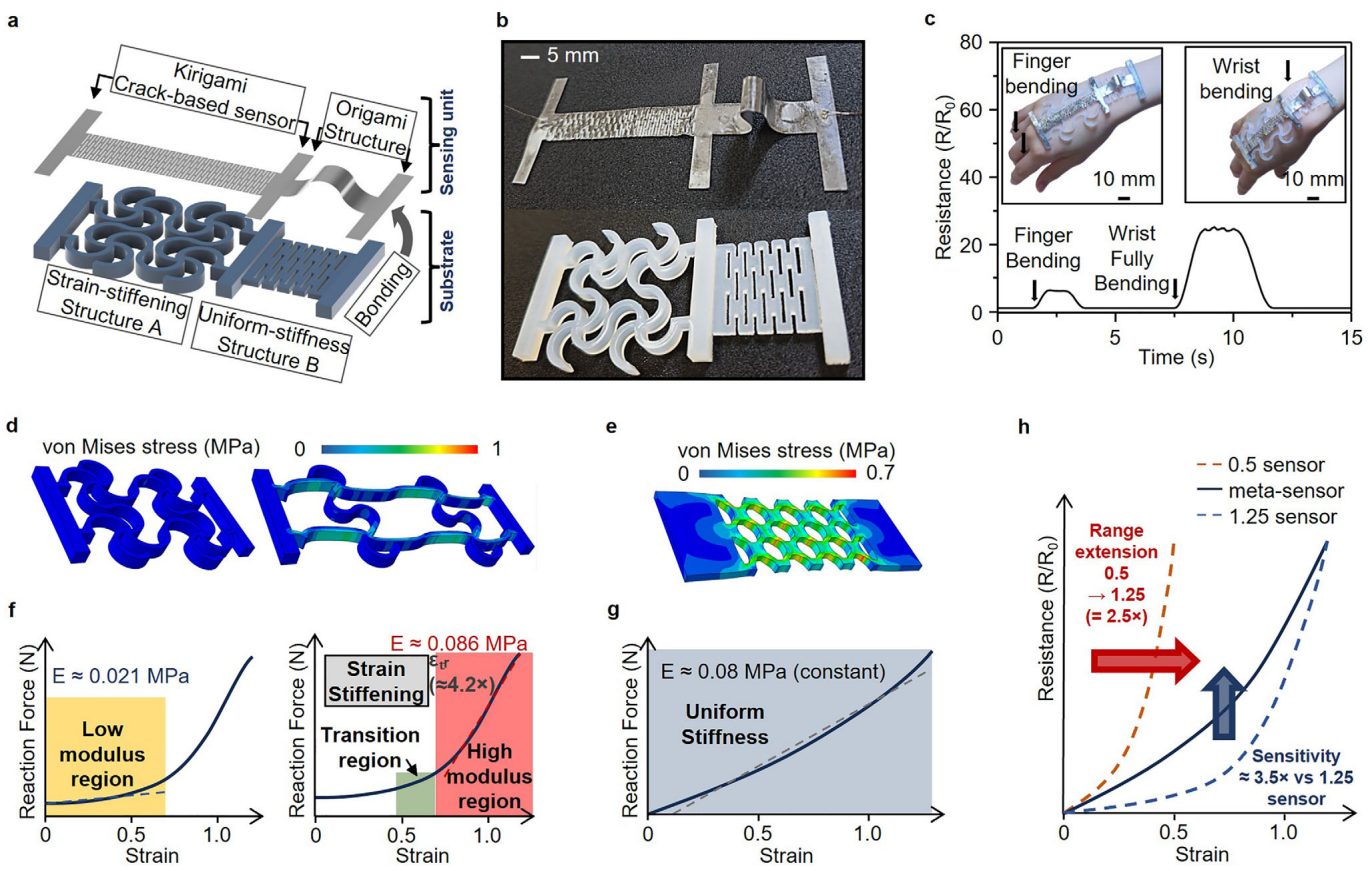
Metamaterial‐based flexible strain sensor platform. (a) Exploded schematic showing the four components: kirigami crack‐based sensing layer, origami interconnect structure, strain‐stiffening structure A, and uniform‐stiffness structure B. (b) Photograph of fabricated components. (c) Resistance–time response of the meta‐sensor on the wrist, detecting both subtle fingers bending and large‐amplitude wrist bending. (d) FEM von Mises stress contours of structure A, undeformed (left) and stretched (right) (0–1 MPa). (e) FEM von Mises stress contour of structure B (0–0.7 MPa). (f) Reaction force–strain of structure A: strain‐stiffening transition from low‐modulus (E ≈ 0.021 MPa) through a transition region to high‐modulus (E ≈ 0.086 MPa), ≈4.2× increase. (g) Reaction force–strain of structure B, constant/uniform stiffness (E ≈ 0.08 MPa). (h) Resistance–strain (*R*/*R*_0_) comparison of the 0.5, 1.25, and meta‐sensor, showing 2.5× range extension (50% → 125%) and ≈3.5× mid‐range sensitivity enhancement.

The integrated sensor (Figure 1a) consists of two mechanically distinct zones. The upper zone houses the active transduction element: a crack‐based resistive sensor patterned in a Kirigami geometry [8], with electrical connections routed through an origami‐folded metal‐coated film [18] that serves as a compliant, low‐resistance interconnect. The lower zone constitutes the metamaterial substrate, composed of two sub‐structures bonded end‐to‐end in a series configuration. The origami interconnect is attached to the metamaterial substrate in a pre‐strained state (125% applied strain), so that the interconnect buckles out‐of‐plane upon relaxation and remains mechanically decoupled from the substrate until the applied strain exceeds 125%; within this range, the crack‐based sensor is the sole source of resistance modulation. The fabricated sensor is shown in Figure 1b.

The crack‐based sensing element exploits the microcrack topology inherent in a sputter‐deposited metallic thin film on a silicone rubber substrate (Figure S1a) [5]. In the undeformed state, crack lips are fully engaged across the film width, providing a large number of parallel conductive pathways and a low initial resistance. As tensile strain is applied, Poisson contraction along the transverse direction forces crack lips to slide and partially disengage; the progressive reduction in conductive bridges produces an approximately exponential increase in resistance with strain, encoded in an exceptionally high gauge factor [5, 6]. A Kirigami pattern of periodic slits distributes the applied strain among both stretching and bending deformation modes of the cut ligaments [8, 9], multiplying the strain range over which the crack lips remain measurably coupled. The morphology of the cracks in the sensing film is confirmed by scanning electron microscopy (SEM), as shown in Figure S1b.

The composite platform — comprising a metamaterial substrate beneath a kirigami crack‐based sensing element — is hereafter referred to as the meta‐sensor. The practical motivation for the meta‐sensor design is illustrated in Figure 1c. When attached on the dorsal wrist [3, 4], a conventional high‐sensitivity sensor resolves the subtle skin deformation induced by individual finger flexion, yet saturates immediately upon full wrist bending. A meta‐sensor, by contrast, captures both subtle finger‐induced skin deformations and the large strains of full wrist bending within a single device.

We also examined robustness to off‐axis wrist motion. Because the meta‐sensor is fundamentally a longitudinal (axial) strain sensor, it is comparatively insensitive to radial and ulnar wrist deviation, which load the device primarily in the transverse and shear directions rather than along the sensing axis; the measured *R*/*R*_0_ response during pure radial/ulnar deviation is small relative to the flexion/extension response, confirming low cross‐axis sensitivity and indicating that the strain‐partitioning and decoupling mechanism remains effective under general wrist motion (Figure S2).

Beyond its response to wrist motion, we also consider the physical form factor of the meta‐sensor with respect to wearability. Wearability is governed not only by overall thickness but, more critically, by whether the device can conform to and follow the curvature and deformation of the body, captured by its bending radius and stretchability; here, the bending radius denotes the smallest inner radius of curvature to which the device can conform without loss of function or mechanical failure. The assembled meta‐sensor conforms to curved surfaces down to an inner bending radius of ∼8 mm while retaining a stretchability of 125% applied strain, keeping it within the deformation window required for conformal skin and body mounting (Figure S3). Although the buckled origami interconnect increases the overall thickness of the meta‐sensor (≈5 mm excluding, and ≈20 mm including, the interconnect) relative to conventional thin‐film crack‐based and kirigami sensors, this added thickness does not impede its practical use as a wearable device. Reducing the device profile, in particular the interconnect height, would nonetheless be an important objective for future work.

The mechanical origin of the strain‐stiffening response of the S‐curved structure (Structure A) is elucidated in Figures 1d,f. In the low‐modulus regime, each unit cell undergoes bending‐dominated deformation along its S‐curved arms (upper left), offering negligible resistance to macroscopic elongation [17]. As the applied global strain approaches the transition threshold, the curved arms progressively straighten and the dominant load‐transfer mechanism shifts from bending to axial stretching, precipitating an abrupt rise in stiffness. The pre‐transition graph (lower left) displays a markedly low initial gradient, whereas the post‐transition graph (lower right, 100% applied strain) shows a substantially steeper slope. The FEM contour computed after the transition (upper right) corroborates this mechanism, showing concentrated stress in the now‐taut ligaments of the fully unfolded structure.

The kirigami structure (Structure B) exhibits different mechanical behavior, shown in Figure 1e,g. Its kirigami cut pattern [8, 9, 19] continuously redistributes the applied load between out‐of‐plane bending of the connecting ligaments and in‐plane stretching, resulting in a spatially homogeneous von Mises stress field throughout the deformation history (top, 50% applied strain). The global reaction force increases proportionally with applied strain, yielding a linear force–strain relationship (bottom graph) and a constant effective modulus across the full operational range. When Structure B is connected in series with Structure A, it provides a stable mechanical reference that allows predictable, tunable strain partitioning between the two sub‐structures.

The combined electromechanical advantage of the meta‐sensor is quantified in Figure 1h. A standalone kirigami crack‐based sensor with a maximum applied strain range of 50% (the ‘0.5 sensor’) delivers high initial sensitivity but saturates at 50% applied strain [5]. The meta‐sensor extends the effective sensing range to 125% applied strain, i.e., 2.5× the original range (from 50% to 125%). Within the pre‐transition regime, the meta‐sensor generates a resistance response approximately 3.5× that of a wide‐range (125%, the ‘1.25 sensor’) kirigami crack‐based sensor over the same strain interval.

### 2.2 Mechanical Characterization of Meta‐Material Structures

**FIGURE 2:**
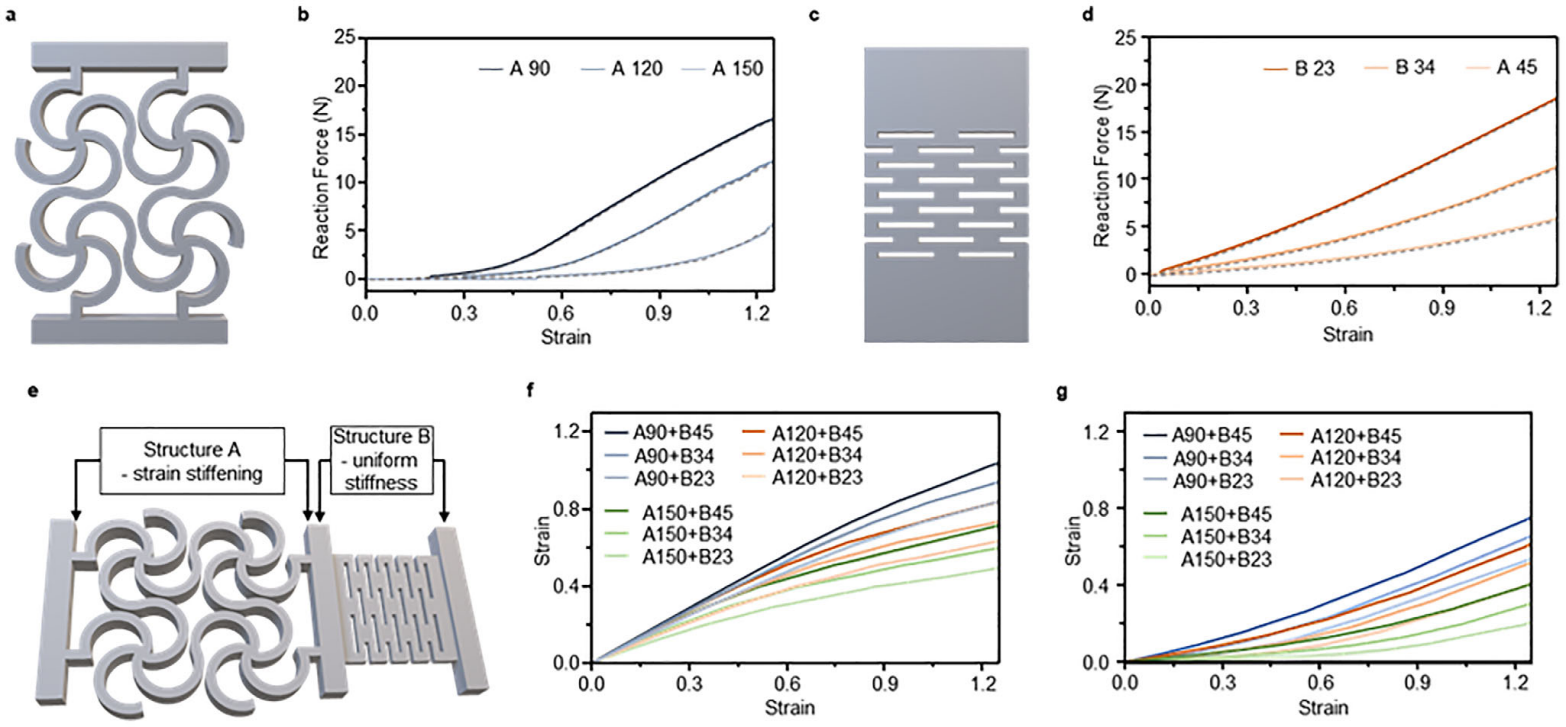
Design and mechanical characterization of the A+B metamaterial platform. (a,b) Geometry and reaction force–strain curves (solid: experiment, dashed: simulation) of structure A variants A150, A120, and A90. (c,d) Geometry and reaction force–strain curves of structure B variants B23, B34, and B45. (e) Rendering of the series‐connected A+B composite assembly. (f,g) Local strain in structure A (f) and structure B (g) vs. total applied strain for all nine A+B combinations, illustrating programmable strain redistribution between the two structures.

Structure A consists of S‐curved mechanical meta‐material [17] and is illustrated in Figure 2a. The geometry is parameterized by the inner radius *r* (=2 mm for all samples), the arc of the inner segment θ_1_, and the arc of the outer segment θ_2_. Three variants — A90, A120, and A150 — span a range of pre‐deformation curvatures with θ_2_ values of 90°, 120°, and 150°, and θ_1_ values of 90°, 80°, and 70° for A90, A120, and A150, respectively (Figure S4).

The reaction force–strain responses of the three Structure A variants are presented in Figure 2b, showing a pronounced bi‐linear characteristic in all specimens. In the pre‐transition regime, the effective elastic modulus of A90, A120, and A150 was approximately 0.021, 0.018, and 0.011 MPa, respectively — three orders of magnitude below the bulk silicone modulus (∼2.28 MPa), reflecting the bending‐dominated deformation of the S‐curved arms. The transition occurred at ≈40% applied strain for A90 and ≈74% for A120, after which the effective elastic modulus rose to approximately 0.086 and 0.082 MPa, respectively — stiffness ratios of 4.2× and 4.5× relative to their pre‐transition values. Structure A150, whose transition strain exceeds the tested range (>125% applied strain), maintained a modulus of 0.011 MPa throughout the measurement window, showing that a larger θ_2_ angle substantially delays the onset of stiffening. FEM simulation results at applied strains of 0%, 40%, and 125% are presented in Figure S5 and show good agreement with the experimental data in Figure 2b [20].

The geometry of Structure B is presented in Figure 2c. B23, B34, and B45 produce progressively lower stiffnesses controlled by the slit‐length parameter c (3, 2, 1.4 mm, respectively), illustrated in Figure S6. Unlike Structure A, all three Structure B specimens exhibit a highly linear force–strain response throughout the full tested range (R^2^ > 0.97 in all cases), with effective elastic moduli of 0.287, 0.163, and 0.081 MPa for B23, B34, and B45, respectively [8, 19]. The modulus of B45 (0.081 MPa) is approximately 3.9× greater than the pre‐transition modulus of A90 (0.021 MPa), ensuring that in the A90+B45 composite, the majority of global strain is concentrated within Structure A prior to its transition. FEM simulation results presented in Figure S7 are in good agreement with the experimental force–strain responses in Figure 2d across all three Structure B variants.

When Structure A and Structure B are joined in series (Figure 2e), the total applied strain partitions between the two sub‐structures according to their instantaneous compliance ratio. Figure 2f,g show the local strain in Structure A and Structure B, respectively, as a function of total composite strain for all nine A–B combinations. In the early deformation regime, the low‐stiffness Structure A accommodates a disproportionately large fraction of the total deformation; for the A150+B45 combination, local Structure A strain reaches approximately 104% at a total applied strain of 125%. Once the transition threshold is passed, its effective modulus exceeds that of Structure B, and additional strain is redirected into Structure B. This redistribution means that at total strains beyond the A‐structure transition, Structure B therefore bears the majority of additional elongation, effectively extending the global strain window without driving the sensor into saturation.

### 2.3 Electrical Characterization of Meta‐Sensors

**FIGURE 3:**
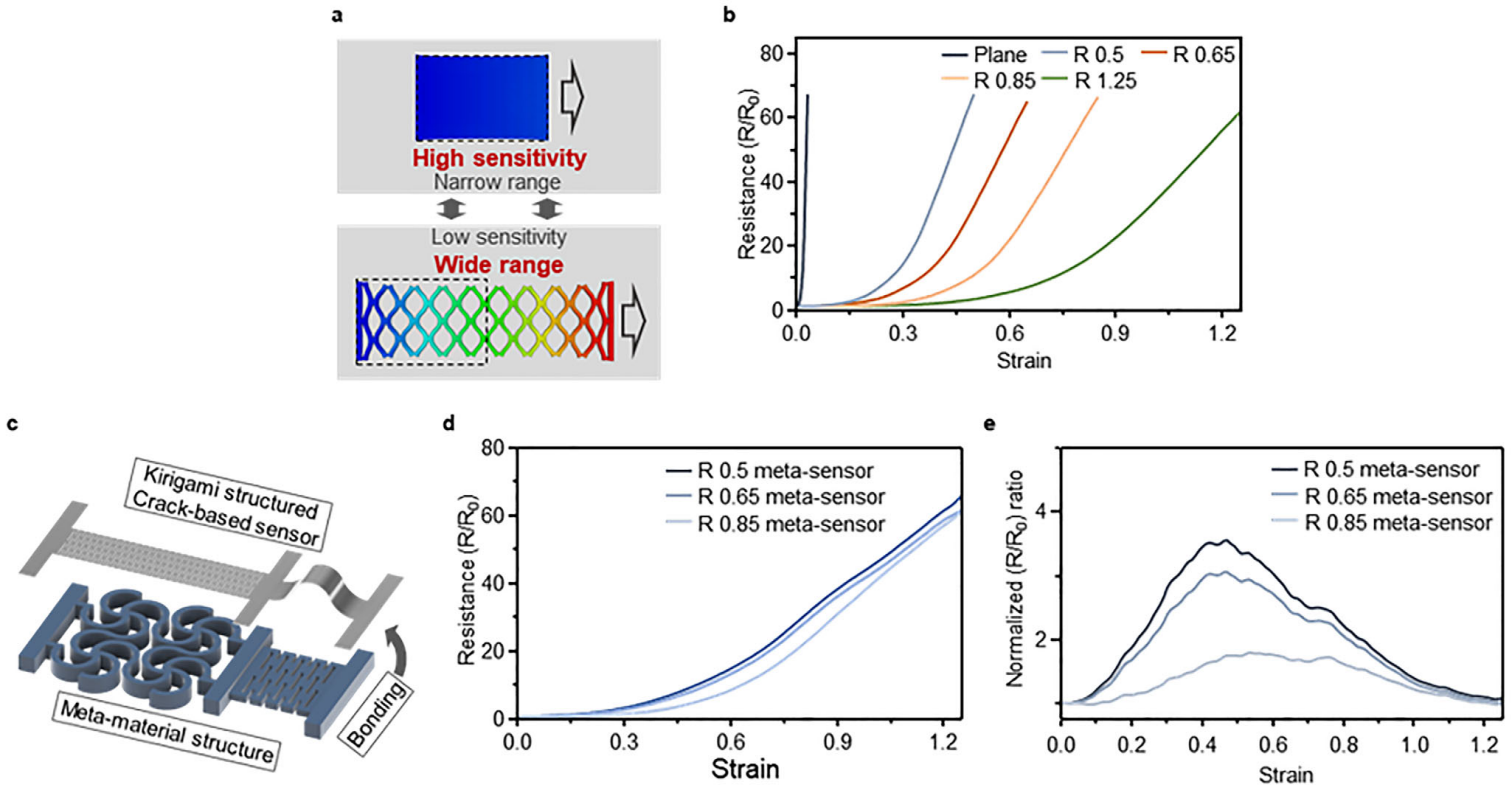
Electrical characterization of kirigami crack‐based sensors and meta‐sensors. (a) Schematic illustrating the fundamental sensitivity–range trade‐off between a high‐sensitivity narrow‐range sensor and a low‐sensitivity wide‐range kirigami sensor. (b) *R*/*R*_0_–strain curves for Plain, R0.5, R0.65, R0.85, and R1.25 sensors. (c) Schematic of the bonding process integrating a kirigami sensor onto the metamaterial structure. (d) *R*/*R*_0_–strain curves of R0.5, R0.65, and R0.85 meta‐sensors, all extending the sensing range to 125% applied strain. (e) Sensitivity ratio of each meta‐sensor normalized by *R*/*R*_0_ of the R1.25 reference, quantifying the sensitivity gain across the full strain range.

Figure 3a illustrates the conceptual distinction between a plain crack‐based sensor — an unpatterned crack sensor bonded directly to the substrate without kirigami slits or a metamaterial substrate, hereafter the ‘Plain’ sensor (high sensitivity, narrow range) [5, 6] and a Kirigami crack‐based sensor (low sensitivity, wide range) [8, 10]. Figure 3b shows measured *R*/*R*_0_ vs. applied strain for one Plain sensor and four Kirigami sensor variants. The four Kirigami variants (R0.5, R0.65, R0.85, and R1.25) share a common unit‐cell geometry parameterized by the slit length *a*, ligament width *b*, and cut spacing *c*, and differ only in *a* — set to 4, 5.2, 6.8, and 10 mm (with *b* = *c* = 1 mm), respectively — so that a longer slit distributes the applied strain over a correspondingly wider range; the unit‐cell design and the four fabricated samples are shown in Figure S8. The Plain sensor saturates at *R*/*R*_0_> 67.25 within ≈3% applied strain. The Kirigami variants progressively extend the operating range: R0.5 reaches *R*/*R*_0_ = 67.3 at 50%; R0.65 reaches *R*/*R*_0_ = 65.3 at 65%; R0.85 reaches *R*/*R*_0_ = 66.3 at 85%; and R1.25 reaches *R*/*R*_0_ = 62.0 at 125% applied strain. Each successive range extension is accompanied by a reduction in sensitivity, consistent with the canonical trade‐off in purely structural approaches [11, 21, 22].

Three meta‐sensors were assembled by bonding the R0.5, R0.65, and R0.85 Kirigami crack‐based sensors to the A90+B45, A120+B45, and A150+B45 metamaterial substrates, respectively (Figure 3c). Among the nine composite configurations in Figure 2f, the three combinations incorporating Structure B45 — A90+B45, A120+B45, and A150+B45 — exhibited the most pronounced nonlinear relationship between global strain and local Structure A strain, as quantified by the nonlinearity index (NL%), defined as the maximum deviation from a linear fit normalized to the full‐scale output. Within each A‐type group, the B45 pairings yielded the highest NL%: 10.39%, 8.55%, and 6.33% for A90+B45, A120+B45, and A150+B45, respectively, owing to the lowest modulus of Structure B45 (0.081 MPa), which maximizes the stiffness contrast between the two sub‐structures.

The resistance–strain responses of the three meta‐sensor configurations are presented in Figure 3d. When bonded to the A90+B45, A120+B45, and A150+B45 composite structures, the effective sensing ranges of the R0.5, R0.65, and R0.85 sensors were each extended to a global strain of 125% — corresponding to range ratios (extended ÷ original range) of 2.5×, 1.9×, and 1.5× for the R0.5, R0.65, and R0.85 meta‐sensors, respectively. The final *R*/*R*_0_ at maximum global strain was 66.1, 61.8, and 60.7 for the three meta‐sensors, closely matching the saturation values of the corresponding standalone sensors (67.3, 65.3, and 66.3). Here we define saturation as the applied strain beyond which *R*/*R*_0_ no longer increases monotonically; because the *R*/*R*_0_ curves in Figure 3d continue to rise at 125%, the meta‐sensors are not saturated at 125% applied strain, which is therefore reported as the characterized (not the limiting) range, and each sensing element operates across its full designed strain window without reaching its detection ceiling.

Figure 3e plots the *R*/*R*_0_ of each meta‐sensor normalized by the contemporaneous *R*/*R*_0_ of the R1.25 sensor at the same global strain. The R0.5 meta‐sensor exhibits a peak normalized ratio of 3.56 at 47% applied strain — that is, its *R*/*R*_0_ is 3.56× that of the R1.25 reference at the same global strain. The R0.65 and R0.85 meta‐sensors peak at ratios of 3.06× and 1.79× the reference at 53% applied strain, respectively.

Although the sensor response is reported throughout as the normalized change *R*/*R*_0_ to enable cross‐device comparison, the absolute resistance levels are also relevant for practical readout. Across devices, the meta‐sensor exhibits a baseline resistance of *R*_0_ = 299.0 ± 5.8 Ω (mean ± s.d., *n* = 20) and a maximum resistance of *R*_max_ = 22.7 ± 0.4 kΩ. These values allow the meta‐sensor to be incorporated as one arm of a Wheatstone bridge whose remaining resistors are chosen close to *R*_0_, so that the bridge is balanced at rest and its differential output—amplified by a low‐power instrumentation amplifier and digitized—scales with the fractional resistance change of the sensor, making the device compatible with compact, battery‐powered wearable acquisition electronics.

Mechanical durability and dynamic performance of the R0.5 meta‐sensor were evaluated in a set of supplementary tests (Figure S9). Cyclic loading to 125% applied strain over ten consecutive cycles (Figure S9a) showed fully reproducible resistance excursions with no baseline drift. A step‐response measurement (Figure S9b) revealed rise and recovery times of 108 and 150 ms, attributable to viscoelastic relaxation of the silicone matrix and sufficient for the temporal requirements of most physiological monitoring applications [23, 24]. An endurance test over 5000 loading cycles (Figure S9c, peak *R*/*R*_0_ recorded at 1,000‐cycle intervals) showed peak *R*/*R*_0_ values ranging from 65.5 to 67.1, with a cycle‐to‐cycle standard deviation below 1.5% and no systematic degradation. Representative stretch–release resistance–time waveforms overlaid at the beginning, middle, and end of the 5000 cycles (Figure S9d) show that the waveform shape is preserved with negligible baseline drift and no crack‐propagation‐ or delamination‐related failure.

### 2.4 Wearable Motion Monitoring Applications

**FIGURE 4:**
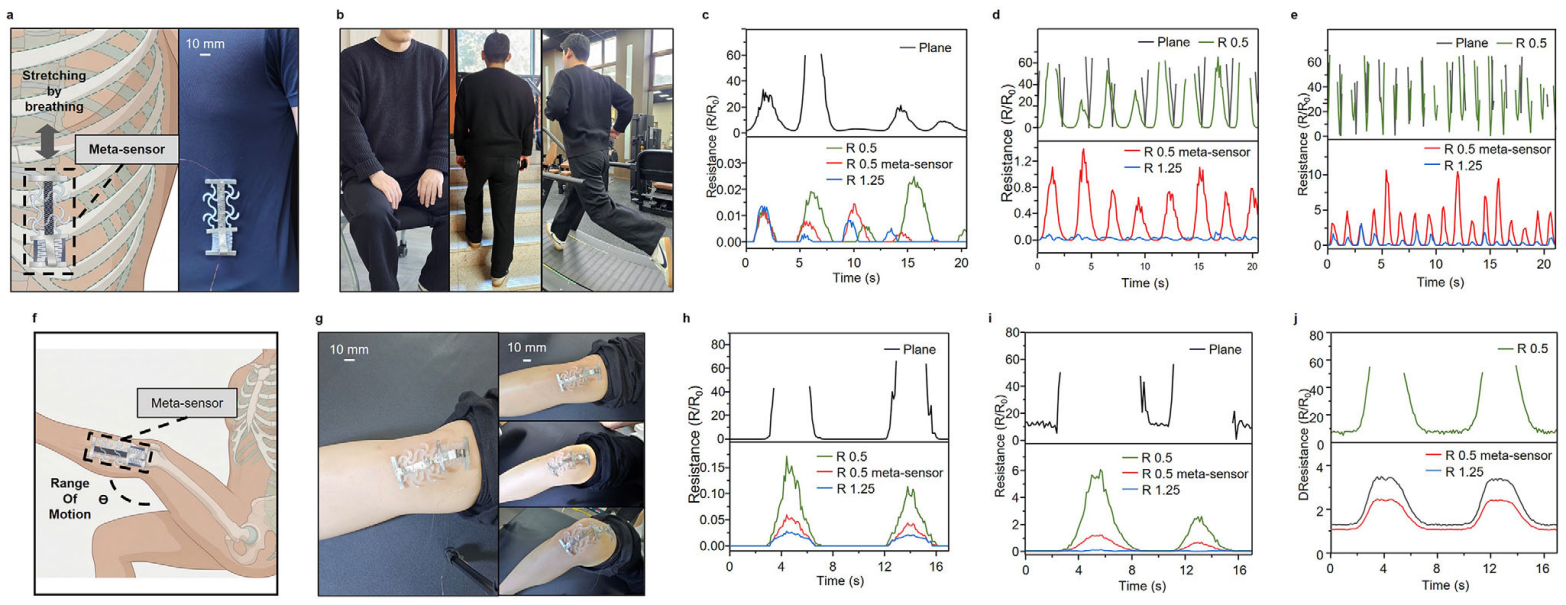
Wearable human motion monitoring demonstrations. (a,b) Respiration monitoring setup at the thoracic rib cage and photographs of three activity states: seated rest, stair climbing, and vigorous running. (c–e) Resistance–time signals from Plain (top) and R0.5/R0.5 meta‐sensor/R1.25 sensors (bottom) during seated rest, stair climbing, and running. (f,g) Knee rehabilitation monitoring setup indicating bending angle θ, and photographs of the sensor on the knee at early, mid, and late rehabilitation stages. (h–j) Resistance–time signals from Plain (top) and R0.5/R0.5 meta‐sensor/R1.25 sensors (bottom) during early, mid, and late rehabilitation stages. Step‐like discontinuities in the Plain‐ and R0.5‐sensor traces indicate over‐range saturation (resistance beyond the instrument's measurement limit).

The meta‐sensor was attached to the lateral thoracic wall at the level of the sixth–8th ribs in the mid‐axillary line, where respiratory excursion imparts cyclically varying skin strain (Figure 4a) [23, 24, 25].

Thoracic skin strain varies substantially across activity levels. At rest, tidal breathing imposes only subtle cyclic deformations; during vigorous exercise, deeper respiratory effort drives significantly larger chest wall excursions at higher rates [24, 25]. A sensor capable of resolving resting tidal breathing would saturate under exercise‐induced hyperpnea, and vice versa. Three conditions were therefore tested: quiet sitting (Figure 4b left), stair climbing (Figure 4b middle), and vigorous running (Figure 4b right).

During quiet sitting, the Plain sensor resolved individual breaths as sharply peaked resistance waveforms with *R*/*R*_0_ amplitudes up to approximately 40. The R0.5 meta‐sensor and R1.25 Kirigami sensor both detected the respiratory rhythm, with lower absolute excursions commensurate with the modest strain amplitude (Figure 4c). During stair climbing (Figure 4d), the increased physical demand produced noticeably larger resistance changes across all sensors. The R0.5 meta‐sensor produced well‐defined periodic signals with higher amplitude relative to the R1.25 sensor, reflecting its sensitivity advantage at moderate strain levels.

During vigorous running (Figure 4e), the Plain sensor and R0.5 sensor exhibited saturation, as the large chest wall strains exceeded their operational ranges. The abrupt discontinuities (step‐like jumps) in the Plain‐ and R0.5‐sensor traces during stair climbing and running mark intervals in which the sensor resistance rose beyond the upper measurement limit of the source‐measure unit; during these intervals the true resistance is off‐scale (over‐range saturation), and the recorded trace is clipped, rather than reflecting a data‐acquisition artifact or intentional truncation. The R0.5 meta‐sensor and the R1.25 sensor remained within the measurable range throughout. The R0.5 meta‐sensor captured each breathing cycle without saturation, while maintaining substantially larger output amplitude than the R1.25 sensor — a combination that neither the high‐sensitivity nor the wide‐range sensor alone could achieve across all three activity conditions [23, 24].

Knee rehabilitation monitoring places a different demand on the sensor: the required ROM increases progressively across recovery stages, from restricted early‐stage exercises (∼30–90°) through moderate mid‐stage ROM (∼60–120°) to near‐full late‐stage ROM (∼150°), while sensitivity must remain sufficient to resolve incremental angle changes at each stage (Figure 4g) [26, 27, 28]. The experimental setup is illustrated in Figure 4f.

In the early rehabilitation stage (Figure 4h), restricted ROM produced small‐amplitude, periodic resistance changes across all sensors. The R0.5 meta‐sensor generated higher‐amplitude waveforms than the R1.25 reference, confirming that the strain concentration mechanism is active even at small joint angles. In the mid‐stage (Figure 4i), the increased ROM drove the standalone R0.5 sensor toward saturation on peak‐angle cycles, while the R0.5 meta‐sensor continued to produce unsaturated, high‐amplitude waveforms that captured the full angular excursion.

In the late‐stage condition (Figure 4j), with ROM approaching ∼150° of flexion, the standalone R0.5 sensor was entirely saturated throughout most of each cycle. The R0.5 meta‐sensor successfully tracked all knee flexion cycles with well‐defined, unsaturated waveforms, enabling quantitative angle reconstruction across the full therapeutic ROM — without sensor substitution or recalibration [26, 27, 28].

## 3 Discussion

The results presented here support the view that the sensitivity–range trade‐off in flexible resistive sensors is not an intrinsic physical limit of the sensing element, but rather a consequence of treating the strain input as a given. By shifting the design focus from the sensor to the substrate, the meta‐material platform removes the coupling between the two‐performance metrics at the source. The nonlinear strain partitioning behavior of the A+B series composite means that the sensor experiences a strain history that is qualitatively different from the boundary strain: concentrated in the pre‐transition regime, and attenuated thereafter. This reshaping of the strain input is what simultaneously yields higher sensitivity and wider range — not any modification of the sensor itself.

The nonlinearity index analysis shows that the stiffness contrast between Structure A and Structure B is the primary lever controlling strain concentration. The B45 configurations consistently produced the most pronounced nonlinear partitioning across all A‐type variants, because the lower modulus of B45 (0.081 MPa) maximizes the compliance ratio prior to the A‐structure transition. This points to a straightforward design rule: for a target strain window and sensitivity profile, one selects the A‐variant to set the transition strain and the B‐variant to set the degree of pre‐transition concentration. The two parameters are largely decoupled, which simplifies the design process.

One aspect that deserves attention is the dependence of the sensitivity enhancement on the choice of sensing element. The 3.56× peak response (i.e., 3.56× the R1.25 reference) reported for the R0.5 meta‐sensor reflects the specific exponential resistance–strain characteristic of the crack‐based sensor; a sensor with a more linear response would show a different enhancement factor, though the operating principle remains unchanged. For sensors with near‐linear characteristics, the primary benefit of the meta‐material substrate would be range extension rather than sensitivity amplification, since sensitivity enhancement scales with the steepness of the sensor's native response curve. This practical nuance should be accounted for when applying the platform to other transducer types.

The wearable application results highlight a practical challenge that is often underweighted in the sensor literature: in real physiological monitoring scenarios, the required strain range and sensitivity are not fixed but vary with the user's activity state or rehabilitation stage. A sensor that performs well under one condition frequently fails under another. The meta‐sensor addresses this mismatch not by calibration or switching between sensors, but by matching the mechanical input transformation to the expected strain envelope of the application through structural parameter selection. This design‐time solution is preferable in wearable contexts where recalibration is impractical.

A further limitation concerns miniaturization: reducing the metamaterial structures to sub‐centimeter scales may be required for certain wrist or finger placements. In the current process, the structures are cast in 3D‐printed molds, and among all dimensions the width of the metamaterial structure—in particular the Structure A width (currently 2 mm)—is the single most fabrication‐limiting feature, because it is the narrowest wall that must be released intact during demolding; with the present molding process, features narrower than ∼2 mm cannot be reliably fabricated. Miniaturization to finger/wrist scale therefore requires a higher‐resolution fabrication process (e.g., high‐resolution or micro‐molding) and rescaling of the structural parameters and is identified as a critical direction for future work. These are tractable engineering problems, and addressing them would substantially broaden the application scope of the platform.

Whether the platform can be scaled to miniaturized geometries, integrated with active electronics, or extended to capacitive and piezoelectric transducers remain open questions; each represents a tractable next step toward practical deployment in continuous wearable monitoring.

## 4 Methods

### 4.1 Fabrication of the Crack‐based Sensing Film

Sensing films were fabricated by sputter‐deposition of a 50 nm platinum (Pt) thin film onto a 30 µm silicone rubber (KEG‐2000‐50‐A/B) substrate. Prior to deposition, silicone rubber was dissolved in a tetrahydrofuran (THF) solution at a 1:2 wt ratio and spin‐coated. The film was solidified at 70°C in a dry oven for 1 h. Kirigami cutting patterns were transferred using fiber laser micromachining (Speed: 1 mm/s, Power: 100%) (MR.CARVE‐M1 Pro, China).

### 4.2 Fabrication of Metamaterial Structures

Structure A and Structure B substrates were fabricated from a platinum‐cured addition silicone elastomer (Shore A 10, 1:1 base‐to‐crosslinker ratio). The two‐component system was mixed at the specified ratio, degassed under vacuum for 20 min, cast into 3D printed molds, and cured at 70°C for 2 h.

### 4.3 Mechanical Testing and Finite‐element Simulation

Uniaxial tensile tests were conducted on a universal testing machine (UTM, GT‐D100S, Korea). For composite A+B structures, local strains were tracked using digital image correlation (DIC). Finite‐element models were constructed in Abaqus/Standard 2022 using hybrid solid elements (C3D8H) for the incompressible hyperelastic matrix [20]. Large‐deformation kinematics (NLGEOM = ON) were enabled throughout, and mesh convergence was confirmed across three refinement levels.

### 4.4 Electrical Measurements

Resistance was measured using a four‐wire configuration with a precision source‐measure unit (PXI‐4071, National Instruments Inc., Austin, TX, USA). Data were acquired at 10 Hz for quasi‐static tests and 100 Hz for dynamic measurements.

## Author Contributions

G.L. designed and fabricated the metamaterial structures and crack‐based sensing elements, performed mechanical tensile testing, and conducted the wearable application experiments, including respiratory and knee rehabilitation monitoring. S.‐W.K. carried out the electrical characterization of the sensors, analyzed the resistance–strain response data, and contributed to the finite element simulations. Y.W.C. conceived the overall meta‐sensor platform concept, supervised the project, and was the primary contributor to writing and revising the manuscript. All authors read and approved the final manuscript.

## Conflicts of Interest

The authors declare no conflicts of interest.

## Supporting information




**Supporting File**: gch270154‐sup‐0001‐SuppMat.docx.

## Data Availability

The data that support the findings of this study are available in the supplementary material of this article.
