# Supplementary material for: Nonlinear Strain Engineering via Meta‐Material Substrates for Flexible Sensors With Simultaneously Enhanced Sensitivity and Wide Sensing Range
Source: Glob Chall. 2026 Sep 8;10(9):e70154. doi: 10.1002/gch2.70154 (PMC13554716; doi:10.1002/gch2.70154)
Supplement: Supplementary file 1 [file GCH2-10-e70154-s001.docx]

**Supplementary Information**

**Nonlinear Strain Engineering via Meta-Material Substrates for Flexible Sensors with Simultaneously Enhanced Sensitivity and Wide Sensing Range**

*Gyeongyeol Lee^1^, Sung-Wook Kim^2^, Yong Whan, Choi^3,*^*

*^1^ Korea Hydro & Nuclear Power Co., LTD.*

*^2^ Department of Electrical and Electronic Engineering, Silla University, Busan 46958, Korea*

*^3^ Department of Aeronautical & Mechanical Engineering, Silla University, Busan 46958, South Korea*

**Fig. S1.** Operating mechanism of the crack-based strain sensor. (a) Schematic crack model represented as interlocking rectangular elements, showing the fully closed low-resistance state at zero strain and the partially reopened high-resistance state under tension, where Poisson contraction transversely re-engages crack lips. (b) SEM image of the crack network formed on the metal-film surface of the fabricated sensor.

**Fig. S2.** Relative resistance (R/R₀) response of the sensor under radial and unar deviation.

**Fig. S3.** The sensor under bending for bending-radius characterization.

**Fig. S4.** Unit cell geometry and key parameters of structure A. Schematics of the spiral unit cell for variants A150 (r = 2 mm, θ₁ = 150°, θ₂ = 70°), A120 (r = 2 mm, θ₁ = 120°, θ₂ = 80°), and A90 (r = 2 mm, θ₁ = 90°, θ₂ = 90°), with arc radius r, inner sweep angle θ₁, and outer sweep angle θ₂ labeled.

**Fig. S5.** FEM tensile simulation results for structure A variants. Von Mises stress contour maps of A150, A120, and A90 (top to bottom) at the undeformed state, within the low-modulus bending-dominated regime, and in the post-transition high-modulus regime (left to right).

**Fig. S6.** Unit cell geometry and key parameters of structure B. Schematics of the kirigami unit cell for variants B23 (a = 9 mm, b = 4 mm, c = 3 mm), B34 (a = 9 mm, b = 4 mm, c = 2 mm), and B45 (a = 9 mm, b = 4 mm, c = 1.4 mm), with total width a, bridge width b, and slit offset c labeled.

**Fig. S7.** FEM tensile simulation results for structure B variants. Von Mises stress contour maps of B23, B34, and B45 (top to bottom) at the undeformed state, intermediate strain with slit opening, and high strain with fully deployed kirigami network (left to right).

**Fig. S8.** Kirigami crack-based sensor design and fabricated samples. (a) Unit cell schematic with labeled parameters a, b, and c for R0.5 (4/1/1 mm), R0.65 (5.2/1/1 mm), R0.85 (6.8/1/1 mm), and R1.25 (10/1/1 mm). (b) Optical photograph of the four fabricated sensor variants (scale bar: 10 mm).

**Fig. S9** Reliability and dynamic performance of the R0.5 meta-sensor. (a) R/R₀ over 10 consecutive stretch–release cycles to 125% strain, confirming repeatable response with negligible baseline drift. (b) Step-response measurement with dashed markers indicating rise and fall transitions, yielding a response time of ~310 ms. (c) Peak R/R₀ recorded at 1,000-cycle intervals up to 5,000 cycles, confirming stable performance without degradation.
